# Thymic Adenocarcinoma Presenting Late as a Large Subcarinal Mass

**DOI:** 10.1016/j.atssr.2023.02.017

**Published:** 2023-03-04

**Authors:** Sangjun Lee, Kwon Joong Na, Samina Park, Sojung Lim, Yoon Kyung Jeon, Hyun Joo Lee, In Kyu Park, Chang Hyun Kang, Young Tae Kim

**Affiliations:** 1 Department of Thoracic and Cardiovascular Surgery, Seoul National University Hospital, Seoul National University College of Medicine, Seoul, Republic of Korea; 2 Cancer Research Institute, Seoul National University College of Medicine, Seoul, Republic of Korea; 3 Department of Pathology, Seoul National University Hospital, Seoul National University College of Medicine, Seoul, Republic of Korea

## Abstract

Thymic malignant neoplasms mostly occur in the anterior mediastinum. Less than 5% occur in the middle or posterior mediastinum. This report describes a large subcarinal mass incidentally discovered during follow-up magnetic resonance imaging for prostate cancer operation. After multidisciplinary discussion, chemotherapy was deemed ineffective, and complete tumor resection through right posterolateral thoracotomy was performed. The operation was uneventful, and the patient recovered smoothly. Immunohistochemistry and pathologic examination revealed thymic adenocarcinoma. Although very rare, thymic adenocarcinomas should be considered in the differential diagnosis of middle mediastinal masses.

Thymic carcinoma is a rare disease that usually arises in the anterior mediastinum. Less than 5% of cases are manifested as middle or posterior mediastinal masses. The most common subtypes are squamous cell carcinoma and lymphepithelioma-like carcinoma.^1^ Regardless of the location, thymic adenocarcinomas are very rare and unlikely to be considered first during the differential diagnosis of mediastinal masses. We report a case of thymic adenocarcinoma manifesting as a large subcarinal mass that was successfully treated with complete surgical resection and diagnosed after thorough immunohistochemistry evaluations.

A 69-year-old man was referred to the pulmonology department for an incidentally discovered large subcarinal mass. He had a history of prostate cancer, and the mass was initially identified on a follow-up magnetic resonance imaging scan. As the mass was only partially covered in the magnetic resonance imaging scan, chest computed tomography (CT) was additionally performed for evaluation. The CT scan revealed a well-defined 6.9-cm (the largest width in the axial view) subcarinal mass with heterogeneous enhancement abutting the left atrium, right lung, and esophagus (Figures 1A, 1B). Positron emission tomography revealed a hypermetabolic mass (maximum standardized uptake value of 49.3) with no evidence of regional or distant metastasis (Figures 1C, 1D). Bronchoscopy and esophageal endoscopy revealed no definite evidence of invasion. The bronchoscopic biopsy findings suggested a “suspicious adenocarcinoma of unknown origin.” As the patient had an underlying diagnosis of prostatic adenocarcinoma, immunohistochemical analysis was performed to rule out metastatic carcinoma. However, the mass was negative for prostate-specific antigen, and the histologic findings indicated that metastatic prostate cancer was unlikely. Furthermore, expression of markers for thymic carcinomas, such as P40, PAX8, c-KIT, and CD5, was not observed in tumor tissue biopsy samples. A multidisciplinary discussion was conducted to establish an appropriate treatment plan. The team agreed that the tumor was less likely to be chemosensitive. Complete resection of the tumor was performed, followed by adjuvant treatment once an adequate diagnosis was achieved from the surgical specimen.

**Figure 1:**
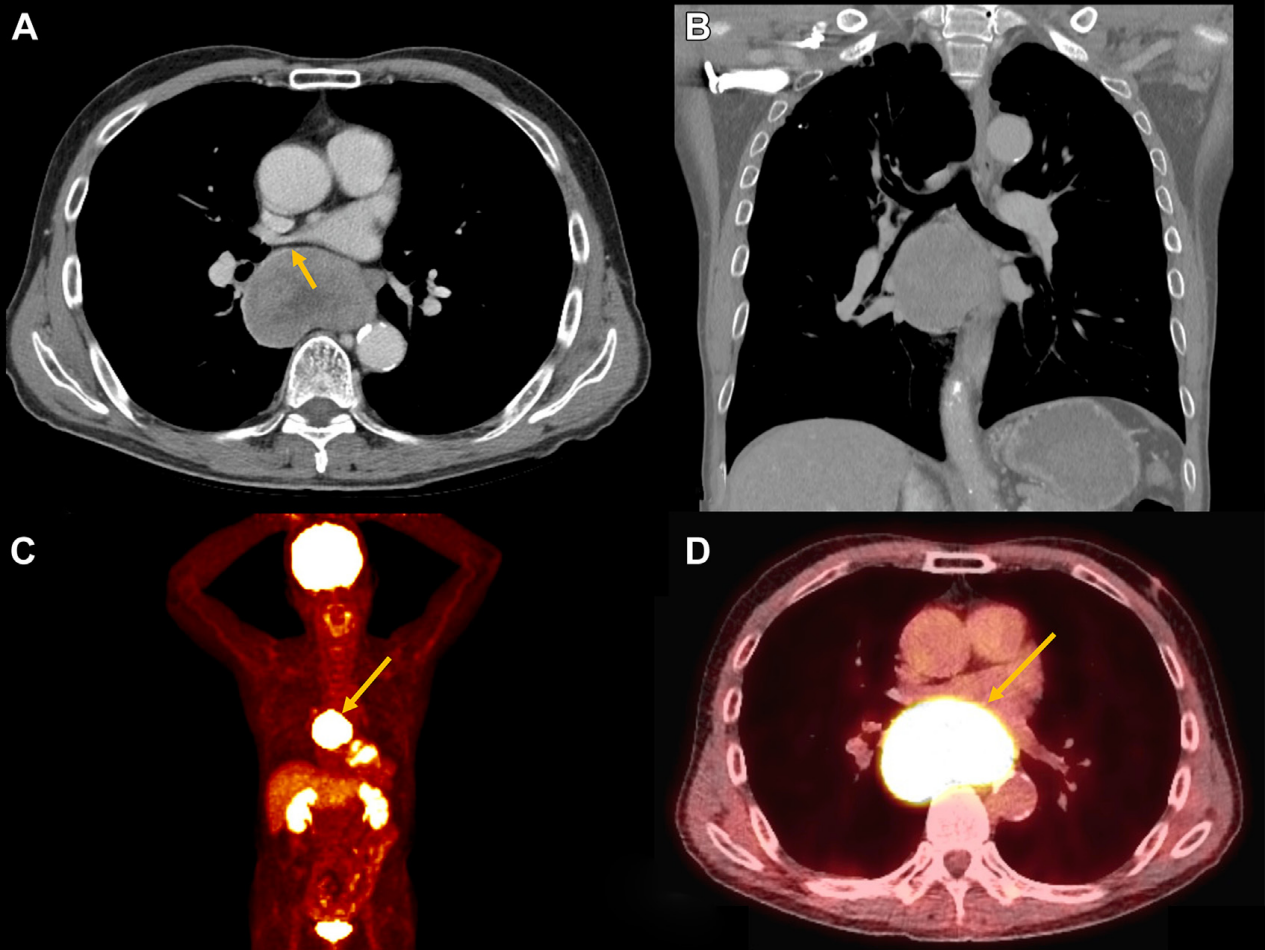
Preoperative computed tomography and fluorodeoxyglucose positron emission tomography findings. (A, B) Heterogeneous subcarinal mass shown abutting the left atrium, right bronchus, and esophagus. (C, D) Fluorodeoxyglucose positron emission tomography scan showing an intensely hypermetabolic subcarinal mass without evidence of distant or regional metastasis. Arrows indicate subcarinal thymic adenocarcinoma.

The right pleural cavity was entered through right posterolateral thoracotomy of the fifth intercostal space. The tumor was densely adherent to the posterior mediastinum and had directly invaded the right lower lobe. No evidence of pleural seeding was observed. The mass was well defined from the aorta, left atrium, esophagus, and left main bronchus; therefore, complete resection of the subcarinal mass with en bloc right lower lobectomy was performed, followed by mediastinal lymph node dissection. The patient underwent concomitant bullectomy of the right upper lobe because of a persistent intraoperative air leak. Postoperatively, the patient underwent a second operation because of a prolonged air leak and was discharged 19 days after the first operation.

Thorough immunohistochemistry analysis of the mass revealed diffuse CK7 and partial CD5 and CDX2 expression. PAX8, TTF1, c-KIT, and napsin A were negative, indicating thymic adenocarcinoma (Figure 2). The tumor had directly invaded the right lower lobe, with no regional lymph node metastases, indicating a pathologic stage of T3 N0 M0, with surgical margins clear of malignant cells. The patient underwent radiation therapy after operation and is currently under outpatient surveillance without any complications or evidence of recurrence for 1 year postoperatively.

**Figure 2:**
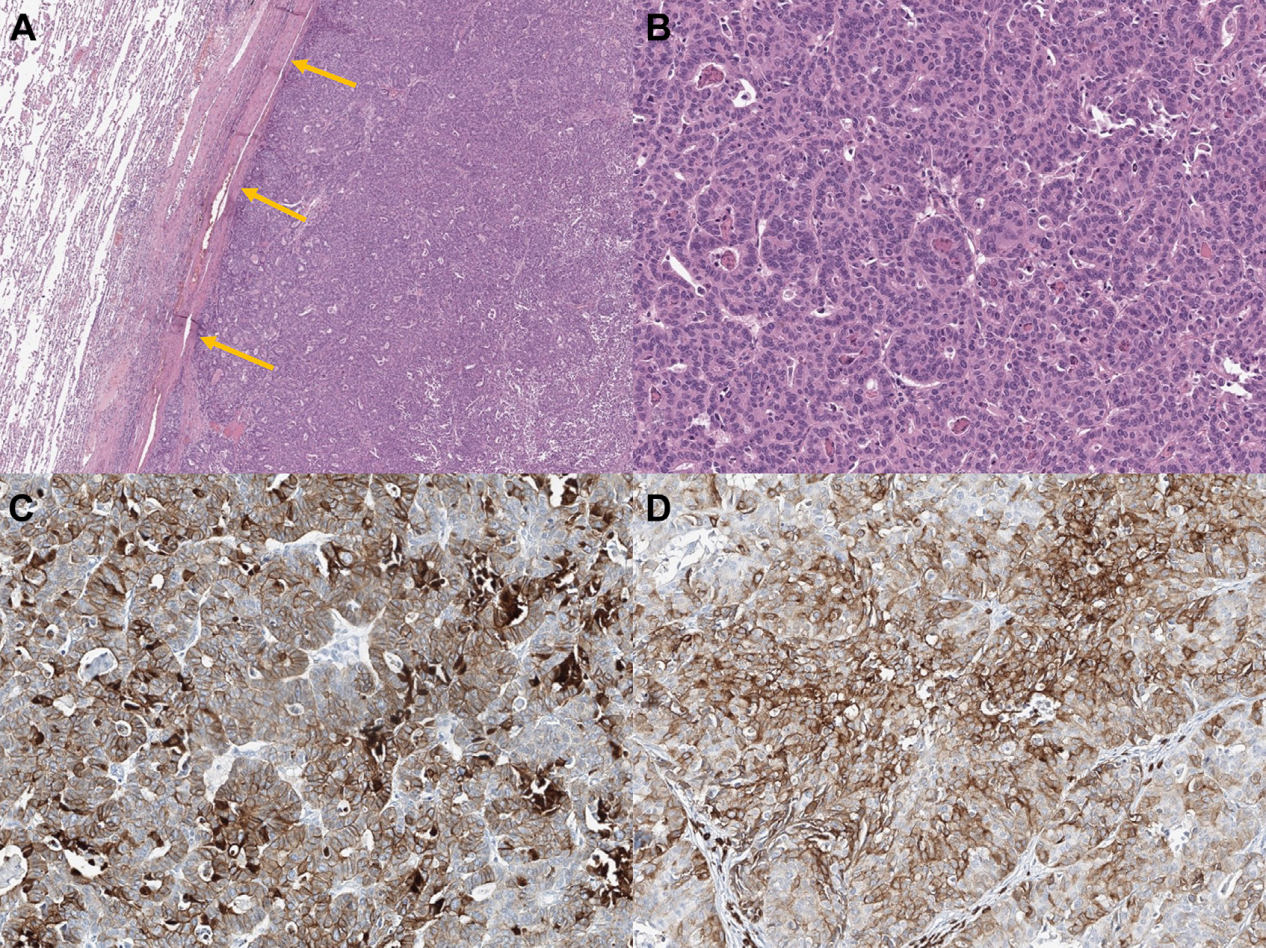
Hematoxylin and eosin (H&E)–stained slides and immunohistochemistry of the surgical specimen. (A) H&E staining (magnification ×40) showing the tumor pushing against the border of the lung parenchyma. (B) H&E staining (magnification ×200) showing glandular architecture. (C) CK7-positive staining. (D) CD5-positive staining. Arrows indicate thymic adenocarcinoma invading lung parenchyma.

## Comment

Thymic adenocarcinomas are rare, with only a few cases reported in the literature. The related symptoms are usually nonspecific, and some cases may be manifested with superior vena cava syndrome or phrenic nerve palsy.^2^ Although it is not possible to generalize the survival rates of all thymic carcinomas to the adenocarcinoma subtype alone because of its rarity, reported cases showed poor survival rates.^2^ Incidental findings, as reported in this case, are not rare, and early detection is usually difficult. The low incidence of thymic adenocarcinomas further obstructs the diagnostic process. Nearly all reported cases of thymic adenocarcinomas were located in the anterior mediastinum, whereas congenital cysts arising from the pericardium or foregut accounted for most middle mediastinal masses.^3^^,^^4^

Tissue confirmation by complete surgical resection remains the best choice for both treatment and diagnosis of thymic tumors. Operation provides superior outcomes compared with radiotherapy or chemotherapy alone in thymic adenocarcinomas.^1^ Thus, preoperative tissue diagnosis, which carries the risk of intrathoracic spillage, must be reserved for unresectable, locally advanced tumors. Radiographic studies provide important information about tumor resectability and can change the entire treatment plan for the disease.

Owing to the unusual location of the tumor, the patient in this case underwent transbronchial needle biopsy preoperatively. Fortunately, the mass had directly invaded the right lower lobe, diminishing the possibility of tumor leakage. This emphasizes the importance of early suspicion during the differential diagnosis of middle mediastinal masses. CT and positron emission tomography scans revealed a well-defined mass with high metabolic activity. These findings are uncommon in thymic carcinomas, which are generally ill-defined, anteriorly located, and usually accompanied by pleural effusions.^3^

Previous studies on thymic adenocarcinoma showed that most cases were positive for cytokeratin 7 and CD5 and negative for PAX8 and TTF1. Most studies emphasized positive findings for CK20 and CDX2, although this case was positive only for CDX2.^4^^,^^5^ The pathology department performed a stepwise investigation following the principle of ruling out the most unlikely differential diagnoses. The possibility of metastatic prostate cancer was excluded by prostate-specific antigen–negative staining, and the diagnosis narrowed toward adenocarcinoma because of the glandular architecture in hematoxylin and eosin staining. After surgical resection and additional histochemical evaluation, the mass was diagnosed as thymic adenocarcinoma.

This is a case of thymic adenocarcinoma occurring in the middle mediastinum. As described in this report, thymic carcinomas, especially adenocarcinomas, generally arise from the middle posterior mediastinum. All possible diagnoses, even those never reported in the literature, should be suspected and considered during the diagnosis of mediastinal masses. Care is needed to avoid overemphasizing the meaning of this report. Primary thymic adenocarcinomas are rare, whereas middle mediastinal thymic adenocarcinomas are even rarer. To reduce the chances of local recurrence and to maximize prognosis, complete resection with negative margins must always be performed.
